# Effect of short-term warming and drought on the methanogenic communities in degraded peatlands in Zoige Plateau

**DOI:** 10.3389/fmicb.2022.880300

**Published:** 2022-10-28

**Authors:** Wei Li, Rui Shi, Lingchen Yuan, Xianli Lan, Defeng Feng, Huai Chen

**Affiliations:** ^1^Yunnan Key Laboratory for Plateau Mountain Ecology and Restoration of Degraded Environments, School of Ecology and Environmental Science, Yunnan University, Kunming, China; ^2^Yunnan Key Laboratory of Plateau Wetland Conservation, Restoration and Ecological Services, Kunming, China; ^3^Key Laboratory of Mountain Ecological Restoration and Bioresource Utilization and Ecological Restoration Biodiversity Conservation Key Laboratory of Sichuan Province, Chengdu Institute of Biology, Chinese Academy of Sciences, Chengdu, China; ^4^Institute of International Rivers and Eco-Security, Yunnan University, Kunming, China; ^5^Institute of Highland Forest Science, Chinese Academy of Forestry, Kunming, China

**Keywords:** wetland, archaea, Qinghai-Tibetan Plateau, climate change, water table

## Abstract

Peatlands in Qinghai-Tibetan are degrading with climate change and human activities. Peatland degradation and climate change affect methane emissions. Methanogens are key functional microbes during methane production; however, knowledge of methanogens in degraded peatlands is lacking. Here, we investigated the effects of short-term (1 year) warming (OTC), drought (20%), and their combination on methanogens in the degraded peatlands on the Zoige Plateau of China via qPCR and clone library analysis. The results showed that *Methanomicrobiales* and *Methanobacteriales* were predominant in all the treatments. Non-metric multidimensional scaling (NMDS) and PERMANOVA analyses showed that the methanogenic community structure among the climate change treatments was not significantly different. The relative abundance of methanogen communities showed insignificant variation among the climate change treatments. The copy number and Shannon diversity of methanogens were significantly different within the climate change treatments, and drought significantly decreased the copy number of methanogens when compared to the control. The Redundancy analysis (RDA) results and correlation analysis showed that the environmental variables measured had no significant effect on methanogenic community structure and Shannon diversity. These results indicate that methanogens are insensitive to short-term climate change in degraded peatlands. This study provides insight into methane emissions from the Zoige Plateau peatlands by focusing on the possible responses of the methanogens to climate-driven changes.

## Introduction

Covering only 3% of the land surface, peatlands contain approximately 30% of the global soil carbon. They are estimated to emit 115–237 Tg methane each year (Christensen et al., 2003), which is a potent greenhouse gas and an important part of the carbon cycle. Climate warming and drought are two important factors that influence methane emissions in peatlands (White et al., 2008). Many studies have shown that warming leads to an accelerated decay rate of organic matter (Melillo et al., 2002; Zhang et al., 2015), consequently increasing methane emissions (Dise, 2009; Dorrepaal et al., 2009; Turetsky et al., 2015). Drought can decrease methane flux (Unger et al., 2021) or increase methane emissions during the Amazonian drought by biomass burning (Saito et al., 2016). Methanogens are the key methane-producing microbes. Understanding the effect of climate change on methanogens, which are methane-producing microorganisms, will help us to better understand the mechanism of peatland carbon emissions, especially in cold regions sensitive to climate change.

The responses of methanogens to warming have been intensively studied in rice field soils (Fey and Conrad, 2000; Wu et al., 2002), peatlands (Metje and Frenzel, 2005; Høj et al., 2007; Metje and Frenzel, 2007; Turetsky et al., 2008; Kim et al., 2012; Cui et al., 2015; Fu et al., 2015), and sediments (Glissman et al., 2004). Some studies have indicated a constant archaeal community structure over a wide temperature range (4–60*^o^*C, 15–20*^o^*C) (Glissman et al., 2004; Metje and Frenzel, 2005, 2007; Cui et al., 2015), while other studies have reported that temperature affects the abundance, diversity, and richness of methanogens (Fey and Conrad, 2000; Wu et al., 2002; Høj et al., 2007; Turetsky et al., 2008; Kim et al., 2012; Fu et al., 2015). This inconsistency may be due to the different warming temperature ranges and site conditions. Moreover, most of these results were obtained from laboratory experiments; hence, *in situ* field experiments are needed to explore the response of methanogens to warming more comprehensively.

In addition, the water table determines the soil oxic/anoxic boundary and redox level (Dinsmore et al., 2009), which can alter the size, structure, and abundance of archaeal communities (Kemnitz et al., 2004; Høj et al., 2006; Tian et al., 2012b; Tian et al., 2015). Drought or a decrease in the water table is known to decrease the abundance of methanogens (Tian et al., 2012b; Urbanová et al., 2013). A study on paddy field soil showed that water-saving practices did not reduce the populations of methanogens, but they moderately influenced the community structure (Watanabe et al., 2013). Another study also revealed that the response of methanogenic communities to water-table drawdowns depended on the initial hydrology (Tian et al., 2015). However, the response of methanogens to short-term soil warming and drought in this degraded peatland is unknown.

The Zoige wetland region (3,400 m a.s.l.), located in the cold Qinghai-Tibetan eastern edge of the Plateau climatic zone, is the primary CH_4_ emission hotspot on the eastern edge of the Qinghai-Tibetan Plateau (Jin et al., 1999). This region is sensitive to climate change (Yang et al., 2014). In this region, climate change is characterized by continuously rising temperatures (Chen et al., 2013) and slightly decreasing precipitation (Hao et al., 2011; Yang et al., 2014). The Zoige peatland in this region is degrading because of climate change, over-grazing, and land reclamation for livestock grazing (Wang et al., 2011; Zhang et al., 2011; Gao et al., 2013; Guo et al., 2013). During the last two decades, several studies on the methanogenic communities in Zoige wetlands using molecular ecology methods have shown that this region contains diverse methanogenic communities (Zhang et al., 2008), the structure and dynamics of which are affected by vegetation type (Tian et al., 2012a), drought (Tian et al., 2012b), water regimes (Tian et al., 2015), and warming (Fu et al., 2015). Fu et al. (2015) observed a warming temperature range of > 10°C under laboratory conditions. However, the response of methanogens to in *situ* warming effects below 3°C in this region is lacking. In addition, the response of these archaeal communities to climate change in degraded peatlands in this region has been less studied (Tian et al., 2012b; Tian et al., 2015). This study aimed to determine the response of methanogens to warming, drought, and their combined effects in a degraded peatland on the Zoige Plateau.

## Materials and methods

### Site description

**FIGURE 1:**
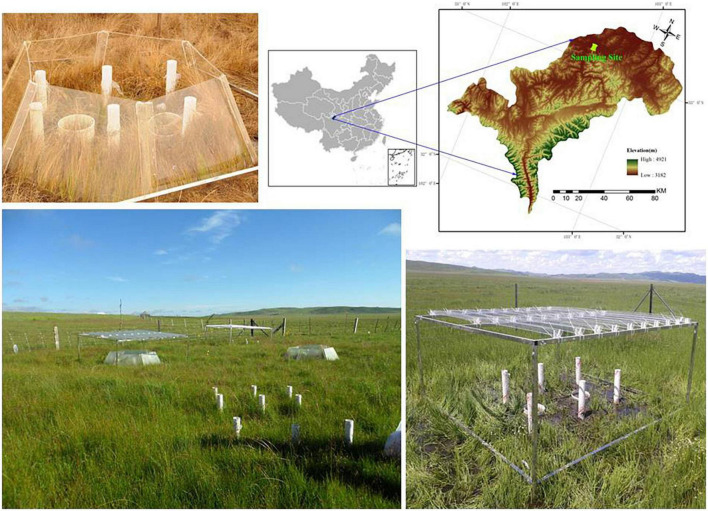
Location of the sampling site and the appearance of the climate change treatments.

The experimental field is located in the Riganqiao Peatland Nature Reserve, a minerotrophic peatland in the Zoige Plateau, Sichuan Province, China (33°06′15″N, 102°39′08″E, 3,471 m a.s.l) (Figure 1). This region is situated in the continental plateau monsoon climate area in the cold temperate zone. The annual average temperature is 1.1*^o^*C, with an extreme minimum temperature of –36*^o^*C and an annual rainfall of 753 mm. Our sampling plots were set up in a degraded peatland that was dry, with an annual average water table of–10 cm and an average soil water content of 61 ± 3.3% (at a depth of 15 cm) during the growing season in 2013. The maximum water-table depth during the growing season is –4.5 ± 1.3 cm, while the minimum during the non-growing season was–30 ± 3.9 cm. For vegetation, *Equisetum ramosissimum* Desf. is the dominant species, while *Caltha palustris* L., *Carex muliensis* hand-Mazz., *Carex meyeriana* Kunth, *Kobresia tibetica* Maximowicz, *and Equisetum ramosissimum* Desf. were mainly observed. The harvested aboveground biomass of this field peatland was 3.60 kg/m^2^ in a 20 cm × 20 cm quadrat (Li et al., 2020).

### Experiment design

The field experiment was conducted in July 2012 and included four treatments: control (ambient temperature and rainfall), temperature change (passive warming with open top chambers), precipitation change (20% drought), and the combined effect (passive warming + 20% drought). Each treatment was performed in triplicate, resulting in 12 treatment plots. The open-top chamber for passive warming was a transparent hexagon PVC chamber that consisted of six equal-sized trapezoids, while the rainout shelter was composed of metal frames with eight V-shaped clear acrylic bands, as described previously (Li et al., 2020).

### Sample collection and soil property analysis

A total of 12 soil cores (0–15 cm) were collected from each treatment in November 2013 using a peat core (8 cm in diameter). At the time of sampling, the soil temperature at the depth of 5 cm and the water table were manually measured using thermometers (DS-1921G-F5#, Maxim/Dallas Semiconductor, Sunnyvale, USA) and rulers, respectively. Samples were then transported on ice to Chengdu Institute of Biology and stored at-20*^o^*C until processing.

Soil cores were homogenized, air-dried, and sieved through a 2-mm diameter mesh for pH, 0.15 mm for total carbon (TC), and total nitrogen (TN) analyses. Approximately 0.05 g of dry soil from each sample core was used to measure TC and TN using a CN Analyzer (Multi N/C the 2100s, Jena, Germany). The soil pH was measured in a soil/water (1:5) suspension. Soil water content was measured using the gravimetric method.

### Deoxyribonucleic acid extraction and *mcrA* gene amplification

DNA was extracted from wet soil (0.5 g) using the Omega E. Z. N. A TM Soil DNA Kit (Omega Bio-tek, USA), following the manufacturer’s instructions. Extraction was performed 3 times and the DNA extracts were pooled. The primer pairs ME1 (5″-GCMATGCARATHGGWATGTC-3″) and ME2 (5″-TCATKGCRTAGTTDGGRTAGT-3″) were used to specifically amplify a 760 bp-long *mcr*A (Hales et al., 1996), which was used to study methanogen communities. The 50 μl reaction mixture contained 1 μL of DNA template, 5 μL of buffer (10 ×), 2 μL of MgCl_2_ (25 mM), 1 μL of deoxynucleoside triphosphates (10 mM), 1 μL of each primer (50 μM), and 2.5 U of Taq DNA polymerase (Takara). Polymerase chain reaction (PCR) conditions were as followers: initial denaturation at 5 min at 94*^o^*C; followed by 30 cycles of 94*^o^*C for 45 s, 50*^o^*C for 45 s, and 72*^o^*C for 2 min, and a final synthesis for 7 min at 72*^o^*C (Bio-rad). The total PCR products were amplified three times and pooled. Amplicons were analyzed on 1% agarose gels with Goldview staining and purified using an Omega PCR purification kit (Omega Bio-tek, USA).

### Clone library, sequencing, and phylogenetic analysis

The purified PCR products were cloned into the pEASY-T1 vector plasmid, according to the manufacturer’s instructions (TransGen, China). Positive clones were sequenced using an ABI 3730xl sequencer (SinoGeno MaxCo., Ltd., China). All *mcr*A sequences were aligned using MEGA 4.0 (Tamura et al., 2007), then assigned to individual operational taxonomic units (OTUs) based on sequence similarity of at least 95% using homology tree analysis in DNAman (LynnonBiosoft, Quebec, Canada). The coverage of the clone libraries was estimated as C = [1-(n/N)] × 100, where *n* is the number of unique clones detected in a sample of size *N* (Good, 1953). The Shannon–Weaver diversity index was calculated as: $H^{\prime }=-\sum_{i=1}^{s}p_{i}\,\ln\left(p_{i}\right)$, where *p*_*i*_ is the proportion of clones belonging to the *i*th OTU and *s* is the total number of OTUs (Shannon, 1996). Phylogenetic trees were constructed using one representative sequence from each OTU and sequences of the reference strains obtained from GenBank. Sequences were aligned using MEGA 4.0, and all gaps were removed. Phylogenetic trees were generated using the neighbor-joining method in MEGA4.0, and the topologies of the resultant trees were evaluated using bootstrap analysis based on 1,000 replicates.

### Real-time polymerase chain reaction

Real-time PCR was performed to quantify the methanogenic archaea 16S rRNA genes using Chromo4 (Bio-Rad, USA) and SYBR Green RealMaster Mix (Tiangen, China). Primer pairs 1106F and 1378R were used to target the 16S rRNA gene of methanogenic archaea (Watanabe et al., 2007). The reaction mix contained 12.5 μL 1 × SYBR Premix, 1 μL of each primer, 1 μL of DNA template, and sterilized distilled water. Further details can be found in Liu et al. (2012). Reactions were prepared in triplicate, and each reaction was run on the same plate with appropriate standards and negative controls. A standard curve was generated using 10-fold dilution series of the linearized plasmid containing the 16S rRNA (1106F and 1378R). The copy number of plasmid/μL was calculated as = (μg plasmid DNA calculated from 260 nm absorption × Avogadro’s number)/(weight of plasmid + insert) (Daniell et al., 2012). The copy number of samples was calculated based on the C_*T*_ value, total volume of DNA (μL), and the weight of fresh soil (g).

### Statistical analysis

Non-metric multidimensional scaling (NMDS) analysis based on Bray–Curtis dissimilarity and permutational multivariate analysis of variance (PERMANOVA) were used to compare the methanogenic archaeal structure among experimental treatments. After testing for normal distribution and homogeneity of variance, one-way ANOVA followed by Tukey’s *post-hoc* test was used to analyze the differences in soil properties, diversity of clone libraries, coverage, Shannon diversity, and the relative abundance of methanogenic communities among the treatments. Redundancy analysis (RDA) was performed to assess the relationship between environmental factors and the abundance of methanogenic communities. Pearson’s correlation analysis was used to examine the correlation between methanogens (relative abundance and Shannon diversity) and environmental variables. Differences were considered statistically significant at *P* < 0.05. All data variations in the means are presented as standard errors (SE).

## Results

### Environmental factors

During the field experiment from July 2012 to November 2013, the monthly average temperatures at 5 cm soil depth were–0.20, 0.76, 0.03, and 2.20*^o^*C in the control, warming, drought, and combined treatment groups, respectively. All treatments had a significant influence on soil pH and TC (Table 1). Warming and the combined effects of warming and drought had a significant influence on soil TN (Table 1). In the four treatments, soil pH, TC, and TN were 5.7–6.0, 19–31, and 1.5–2.2%, respectively, all with the maximum in warming treatment and minimum in the control (Table 1). Climate change treatments had no significant effect on soil water content, which ranged from 60 to 63% (Table 1).

**TABLE 1:** Soil property and diversity analysis of clone libraries under climate change treatments.

	CK	W	R	W + R
5 cm soil temperature (*^o^*C)	–0.20	0.76	0.03	2.20
Soil water content (%)	63 ± 3.8a	61 ± 3.3a	60 ± 4.1a	62 ± 3.1a
pH	5.7 ± 0.03b	6.0 ± 0.03a	5.8 ± 0.09c	5.8 ± 0.01cd
Total carbon (%)	19 ± 0.04b	31 ± 0.31a	26 ± 0.15c	27 ± 0.24ac
Total nitrogen (%)	1.5 ± 0.00b	2.2 ± 0.02a	1.7 ± 0.02bc	1.8 ± 0.00cd
No. of positive clones	52 ± 2.0a	76 ± 3.0c	79 ± 6.0bc	79 ± 2.0dc
Total No. of OTU	15 ± 1.0a	21 ± 2.0b	18 ± 2.0ab	17 ± 1.0ab
Coverage (%)	89 ± 1.7a	91 ± 1.4a	92 ± 0.84a	89 ± 2.2a
Shannon’s diversity index	2.3 ± 0.12ab	2.6 ± 0.10b	2.4 ± 0.11ab	2.3 ± 0.06a

CK, Control (no warming or drought); W, warming; R, 20% drought; W + R, warming +20% drought. *n* = 3, values = mean + SE. Different letters in the same row indicate significant differences at the level of *p* < 0.05.

### Clone library and phylogenetic analysis

**FIGURE 2:**
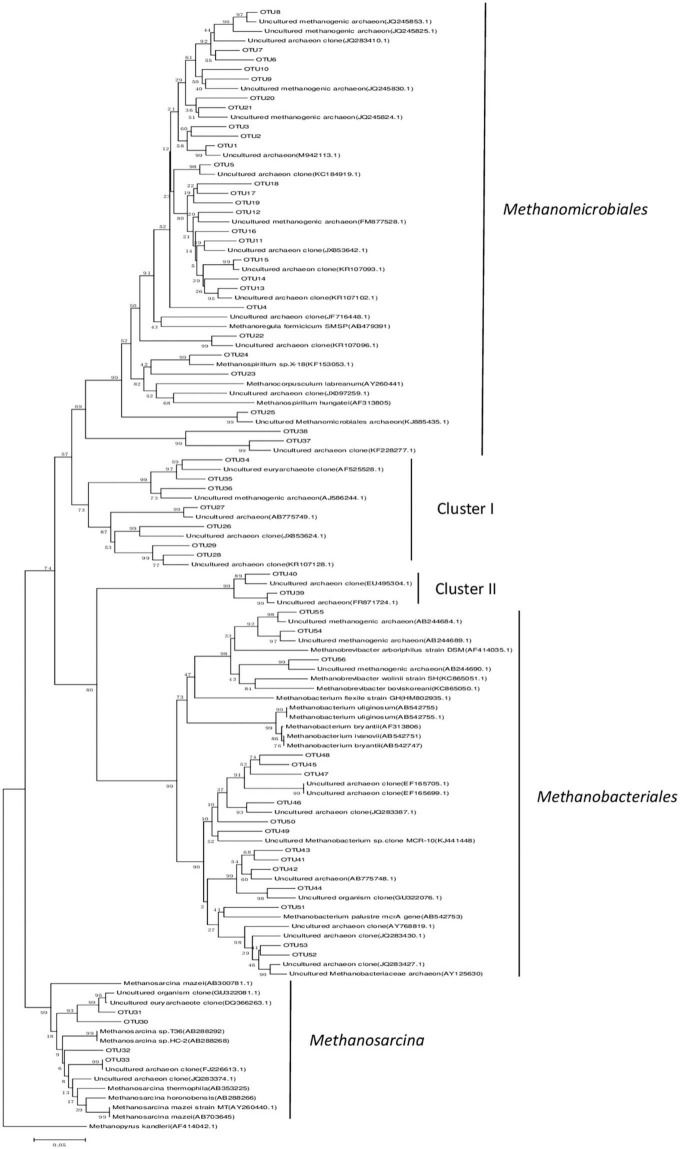
Phylogenetic tree based on the *mcr*A gene clone sequences. Sequences obtained from peatland libraries are designated as OTUs. The tree was constructed using *P*-distance matrix analysis. GenBank accession numbers are indicated for all sequences. *Methanopyrus kandleri* was used as an outgroup. The scale bar indicates a 5% sequence divergence.

A total of 859 positive clones were sequenced and then grouped based on 95% similarity using homology tree analysis in DNAman. Totally 56 OTUs were observed, wherein each group was considered one OTU (Supplementary Figure 1). The percentages of coverage of the clone libraries ranged from 89 to 92% (Table 1), and the richness curves are shown in (Supplementary Figure 2). Phylogenetic analysis indicated that all sequences belonged to *Methanomicrobiales*, *Methanobacteriales*, and *Methanosarcinales*, except for the unknown Cluster I and Cluster II (Figure 2 and Table 2). *Methanomicrobiales* was the dominant taxon (Table 2).

**TABLE 2:** Composition of the methanogenic communities in the four treatments.

Treatment	CK	W	R	W + R
*Methanomicrobiales* (order) (%)	60 ± 3.3	50 ± 7.5	60 ± 4.0	53 ± 2.4
*Methanobacteriaceae* (family) (%)	18 ± 7.8	10 ± 1.5	20 ± 6.0	14 ± 6.2
*Methanobrevibacter* (genus) (%)	0.53 ± 0.53	0.83 ± 0.83	0.00 ± 0.00	1.9 ± 1.9
*Methanosarcina* (genus) (%)	0.00 ± 0.00	1.1 ± 1.1	1.8 ± 0.95	2.3 ± 1.3
Cluster I (%)	9.7 ± 4.0	27 ± 7.7	9.1 ± 2.2	18 ± 8.1
Cluster II (%)	0.56 ± 0.56	2.0 ± 1.1	0.36 ± 0.36	0.00 ± 0.00

CK, Control (no warming or drought); W, warming; R, 20% drought; W + R, warming +20% drought. *n* = 3.

### Effects of warming and drought on methanogenic community

**FIGURE 3:**
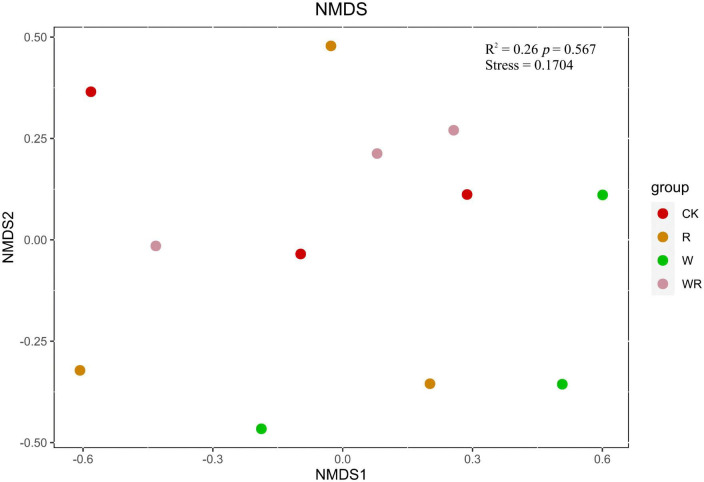
The NMDS plot of methanogenic communities based on Bray–Curtis distance. CK, Control (no warming or drought); W, warming; R, 20% drought; WR, warming + 20% drought.

NMDS analysis showed that the methanogenic community structures among the climate change treatments could not be divided (Figure 3). PERMANOVA also confirmed that the methanogenic community in climate change treatments did not differ significantly (*R*^2^ = 0.26, *P* = 0.57) (Figure 3). The methanogenic communities were dominated by *Methanomicrobiales*, and the microbial order remained similar in all four treatment groups (Table 2). Eleven dominant OTUs and methanogen groups were selected for the analysis of the response of methanogenic communities to climate change. The result showed that warming, drought, or their combination did not have a significant effect on the relative abundance of methanogenic communities (Tables 2, 3). The Shannon diversity index ranged from 2.3 to 2.6 in all four treatments (Table 1), and warming, drought, or their combination did not have a significant effect on the Shannon diversity index when compared to the control (Table 1).

**TABLE 3:** ANOVA results on the response of methanogens to different climate change treatments (*p*-values as compared with control).

	W	R	W + R
*Methanomicrobiales* (order)	0.27	0.97	0.11
*Methanobacteriaceae* (family)	0.36	0.83	0.72
*Methanobrevibacter* (genus)	0.77	0.37	0.53
*Methanosarcina* (genus)	0.37	0.13	0.16
Cluster I	0.11	0.90	0.39
Cluster II	0.14	0.37	0.37

W, warming; R, 20% drought; W + R, warming + 20% drought.

**FIGURE 4:**
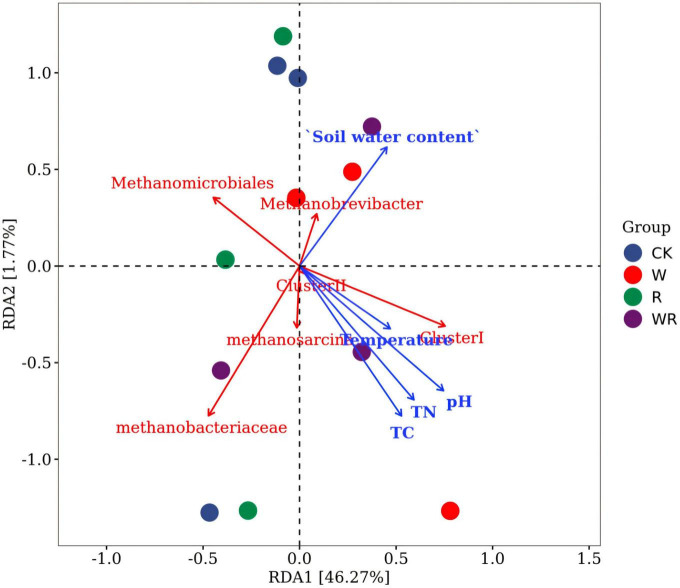
RDA plot of methanogenic communities in relation to environmental factors. CK, Control (no warming or drought); W, warming; R, 20% drought; WR, warming + 20% drought.

The RDA results showed that none of the environmental variables had a significant effect on methanogenic community structure (Figure 4). Further correlation analysis showed that OTU 41 (*Methanobacteriaceae* sp.) was significantly and negatively correlated with TC (*R* = –0.621, *p* < 0.05) and pH (*R* = –0.680, *p* < 0.05). The other OTUs were not significantly correlated with any environmental variable (Supplementary Table 1). At the classified methanogen level, none of the methanogens had a significant relationship with environmental variables. However, Cluster I was significantly and positively related to the TC (*R* = 0.606, *p* < 0.05) and pH (*R* = 0.647, *p* < 0.05) (Supplementary Table 1). The Shannon diversity index of methanogens had no significant relationship with environmental variables (Supplementary Table 1).

### Effects of warming and drought on methanogen abundance

**FIGURE 5:**
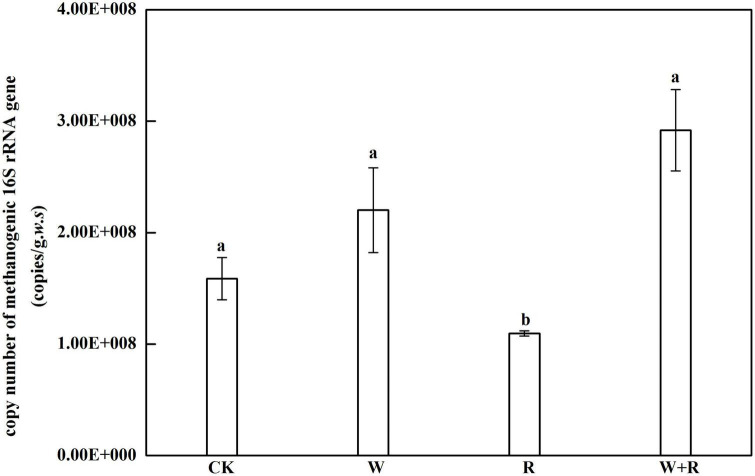
Abundance of methanogens is represented by 16S rRNA genes. Data points show the mean and standard error. Lowercase letters indicate a significant difference at *P* < 0.05.

The abundance of soil methanogens was directly represented by 16S rRNA copy number determined using real-time PCR, and it ranged from 1.1 × 10^8^ to 2.2 × 10^8^ copies/[gram. Fresh soil] (Figure 5). The 16S rRNA copy number in the warming, drought, and combined treatment groups were not significantly different from that of the control group, whereas the drought group significantly decreased the copy numbers of methanogens (Figure 5).

## Discussion

### Methanogenic communities

Hydrogenotrophic methanogens, *Methanomicrobiales* and *Methanobacteriales*, are relatively tolerant to soil drought and aeration (Høj et al., 2006). Previous studies have reported that *Methanomicrobiales* are dominant in dry peatland samples (Galand et al., 2002; Galand et al., 2003; Juottonen et al., 2005; Sun et al., 2012). In our study, the annual average water table of-10 cm may have led to the dominance of *Methanomicrobiales* and *Methanobacteriales* (Høj et al., 2006). This result indicates that hydrogenotrophic methanogenesis may be the main pathway in dry degraded peatland. Our results are consistent with other studies on Zoige dry wetland conditions, which indicated the dominance of hydrogenotrophic methanogenesis (Tian et al., 2012b,Tian et al., 2015). Low water-table (maximum = –4.5 ± 1.3 cm and minimum = –30 ± 3.9 cm) leading to higher soil oxidation-reduction potential that is favorable to hydrogenotrophic methanogenesis (Høj et al., 2006) may have induced hydrogenotrophic methanogenesis. This result was also in accordance with those of previous studies on unsubmerged peatlands with a low water-table, wherein hydrogenotrophic methanogenesis was predominant in the upper part of the Salmisuo fen (-6 cm water table) (Galand et al., 2002) and in a continental bog (water table close to the surface) (Yavitt et al., 2006). Moreover, hydrogenotrophic methanogenesis was the dominant pathway for CH_4_ production in acidic bog peat (pH < 5) (Williams and Crawford, 1984; Lansdown et al., 1992; Horn et al., 2003; Kotsyurbenko et al., 2007). While our study site had a pH of 5.7–6.0, hydrogenotrophic methanogenesis could also account for the CH_4_ production, suggesting that pH may not be the main factor in the methanogenesis pathway. *Methanosarcina* (genus) uses all three known metabolic pathways for methanogenesis (Welte and Deppenmeier, 2011). Therefore, the low relative abundance of *Methanosarcina* (Genus) in our study indicates the presence of aceticlastic and methylotrophic methanogenesis. Incubation experiments are needed to obtain direct evidence of the actual methanogenesis pathways at this site in future.

### Effect of climate change on methanogens

The NMDS plot and PERMANOVA analysis showed that methanogenic communities among the climate change treatments were not significantly different. Climate change treatments also did not significantly affect the relative abundance of the methanogenic communities. These results are consistent with those of previous studies (Metje and Frenzel, 2005, 2007; Cui et al., 2015). In this study, *Methanomicrobiales*, which are tolerant to soil drought and aeration, were dominant in all treatments. This result indicates that a small magnitude of warming was not enough to affect methanogens in the degraded peatland with a low water-table. The effect of warming on methanogens may not stand alone and is related to soil factors, such as initial soil hydrology. The response of archaeal communities to water regimes under simulated warming and drought conditions depends on the initial hydrology (Tian et al., 2015), and the effect of short-term warming on anaerobic methanogens was probably much less than that of the low water table in the degraded peatland. Thus, the key factor affecting methanogens in the degraded peatland may be the water table rather than increased temperature.

Furthermore, drought had on significant effect on relative abundance and community structure of methanogens. Our results were consistent with those of previous studies that also revealed that even some methanogenic populations can be ubiquitous or can survive in oxygen-containing atmospheres (Peters and Conrad, 1995; Kruger et al., 2005; Angel et al., 2012). Long-term drainage would make the microbial community composition in different peatland types similar (Urbanová and Barta, 2016), and the presence of dominant *Methanomicrobiales* in all experimental treatments may be caused by the long term initial low water table in this degraded peatland. Additionally, the water table is a key factor for anaerobic methanogens. Compared with the low water-table in the degraded peatland, the effect of the small amplitude of drought (20%) was probably too weak to disrupt the methanogenic conditions determined by the initial soil water-table. A 20% drought treatment is below the threshold for methanogenic community change in the degraded peatland.

The combined effect of warming and drought had a significant effect on soil bacteria (Zhang et al., 2016); however, we obtained inconsistent results. The different ranges of warming and drought, as well as the initial soil hydrology, may have caused such inconsistencies. Methanogens are a group of functional microorganisms that play key roles in methane production. The insignificant effect of short-term warming and drought on methanogenic communities indicated that these conditions were probably not the main factors influencing methane production in the degraded peatlands on the Zoige Plateau.

This study performed short-term (1 year) warming and drought treatments, and our results only included an integrated view of events occurring at the end of the season. Varying conditions over the growing season may produce different results. Therefore, long-term studies on spatial and temporal variations are needed to reveal the effect of climate change on methanogens in this degraded peatland. Nevertheless, the initial soil water-table is an important factor in degraded peatlands for future model development of the methane cycle.

## Data availability statement

The original contributions presented in this study are included in the article/Supplementary material, further inquiries can be directed to the corresponding author/s.

## Author contributions

HC and WL conceived and designed the study. WL collected the data and contributed field sampling. WL, RS, LY, XL, and DF performed the analysis. RS participated drafting the work. WL and DF wrote and revised the manuscript. HC reviewed and edited the manuscript. All authors contributed to the article and approved the submitted version.

## Supplementary material

Supplementary Figure 1 Average relative abundance of methanogens (OTUs) from clone libraries based on 95% sequence similarity. CK, Control (no warming or drought); W, warming; R, 20% drought; W + R: warming + 20% drought.

Supplementary Figure 2 OTU richness curves of nine *mcrA* clone libraries. Curves were generated using rarefaction calculation. CK, Control (no warming or drought); W, warming; R, 20% drought; W + R, warming + 20% drought.
