# Supplementary material for: Effect of short-term warming and drought on the methanogenic communities in degraded peatlands in Zoige Plateau
Source: Front Microbiol. 2022 Oct 28;13:880300. doi: 10.3389/fmicb.2022.880300 (PMC9650419; doi:10.3389/fmicb.2022.880300)
Supplement: Supplementary Figure 1 — Average relative abundance of methanogens (OTUs) from clone libraries based on 95% sequence similarity. CK, Control (no warming or drought); W, warming; R, 20% drought; W + R: warming + 20% drought. [file Data_Sheet_1.pdf]

Fig.S1

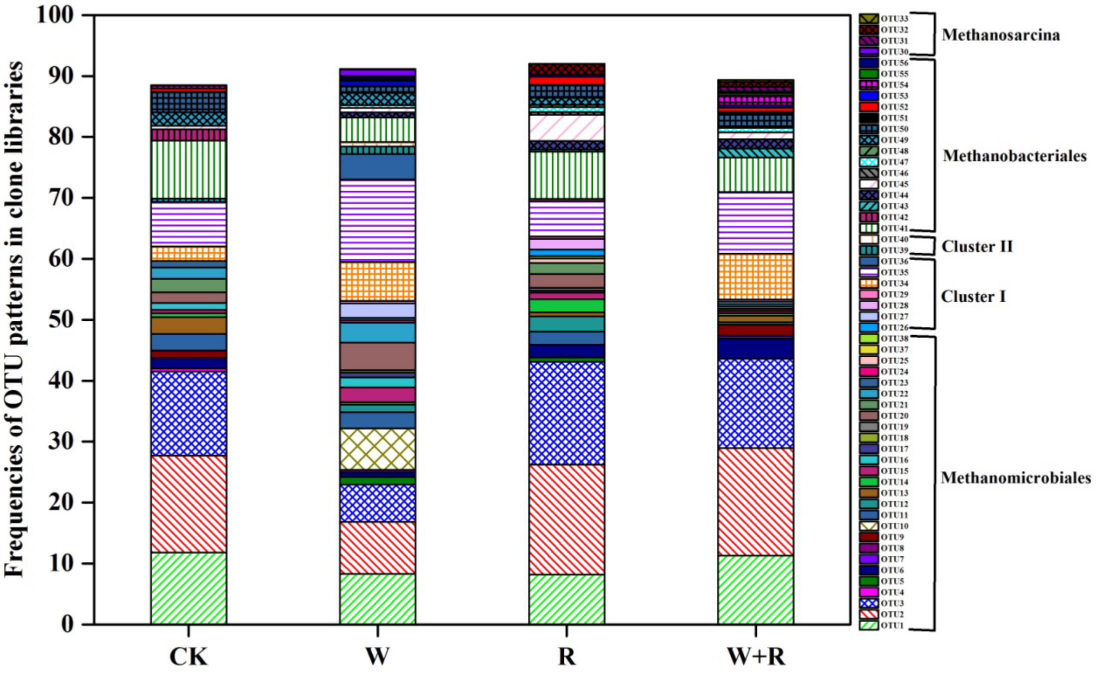

Frequencies of OTU patterns in clone libraries
0
10
20
30
40
50
60
70
80
90
100
CK
W
R
W+R
OTU33
OTU32
OTU31
OTU30
OTU56
OTU55
OTU54
OTU53
OTU52
OTU51
OTU50
OTU49
OTU48
OTU47
OTU46
OTU45
OTU44
OTU43
OTU42
OTU41
OTU40
OTU39
OTU36
OTU35
OTU34
OTU29
OTU28
OTU27
OTU26
OTU38
OTU37
OTU25
OTU24
OTU23
OTU22
OTU21
OTU20
OTU19
OTU18
OTU17
OTU16
OTU15
OTU14
OTU13
OTU12
OTU11
OTU10
OTU9
OTU8
OTU7
OTU6
OTU5
OTU4
OTU3
OTU2
OTU1
Methanosarcina
Methanobacteriales
Cluster II
Cluster I
Methanomicrobiales

Fig.S2

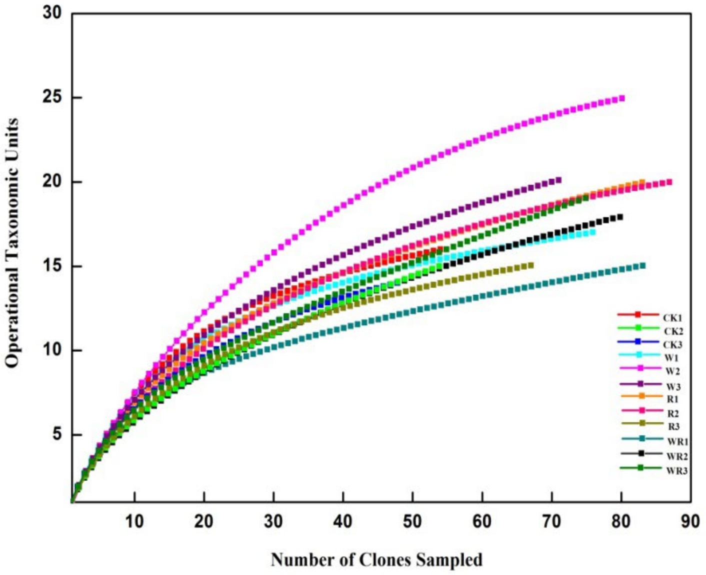
Operational Taxonomic Units
Number of Clones Sampled
30
25
20
15
10
5
10
20
30
40
50
60
70
80
90
CK1
CK2
CK3
W1
W2
W3
R1
R2
R3
WR1
WR2
WR3

Table S1 Correlations of selected 11 OTUs, methanogenic communities and Shannon diversity with environmental variables.

Environmental Variable	Total Carbon	Total Nitrogen	pH	Soil Temperature	Soil Water Content
OTU1	-0.141	-0.156	-0.186	-0.013	0.199
OTU2	-0.087	-0.309	-0. 420	0.002	0.349
OTU3	-0.150	-0.223	-0.094	-0.076	-0.116
OTU6	0.116	0.131	-0.071	0.387	0.297
OTU14	0.065	-0.023	-0.026	-0.186	-0.189
OTU15	0.227	0.221	0.168	-0.008	0.026
OTU35	0.345	0.367	0.496	0.276	-0.010
OTU41	-0.621*	-0.534	-0.680*	-0.381	0.240
OTU45	0.092	0.111	-0.013	0.018	-0.113
OTU50	-0.258	-0.253	-0.230	-0.332	-0.309
OTU52	-0.056	-0.187	-0.285	-0.082	0.131
Methanomicrobiales	-0.314	-0.455	-0.550	-0.509	0.249
Methanobacteriaceae	-0.197	-0.103	-0.256	-0.286	-0.201
Methanobrevibacter	0.071	0.045	0.216	0.211	0.311
Methanoscarnica	0.452	0.286	0.105	0.463	-0.013
ClusterI	0.606*	0.512	0.647*	0.444	-0.196
ClusterII	0.111	0.266	0.157	-0.075	0.118
Shannon diversity	0.230	0.297	0.465	-0.172	-0.127

* Significant at $p < 0.05$.
